# Machine learning prediction of Gleason grade group upgrade between in-bore biopsy and radical prostatectomy pathology

**DOI:** 10.1038/s41598-024-56415-5

**Published:** 2024-03-11

**Authors:** Kaan Ozbozduman, Irem Loc, Selahattin Durmaz, Duygu Atasoy, Mert Kilic, Hakan Yildirim, Tarik Esen, Metin Vural, M. Burcin Unlu

**Affiliations:** 1 Bogazici University Physics Department, Istanbul, Turkey; 2 Department of Radiology, Istanbul Faculty of Medicine, Istanbul University, Istanbul, Turkey; 3 Department of Radiology, University of Koc School of Medicine, Istanbul, Turkey; 4 Department of Urology, VKF American Hospital, Istanbul, Turkey; 5 Department of Radiology, VKF American Hospital, Istanbul, Turkey; 6 Department of Urology, University of Koc School of Medicine, Istanbul, Turkey; 7 Faculty of Engineering, Ozyegin University, Istanbul, Turkey; 8 Faculty of Aviation and Aeronautical Sciences Ozyegin University, Istanbul, Turkey

**Keywords:** Prostate cancer, Cancer imaging

## Abstract

This study aimed to enhance the accuracy of Gleason grade group (GG) upgrade prediction in prostate cancer (PCa) patients who underwent MRI-guided in-bore biopsy (MRGB) and radical prostatectomy (RP) through a combined analysis of prebiopsy and MRGB clinical data. A retrospective analysis of 95 patients with prostate cancer diagnosed by MRGB was conducted where all patients had undergone RP. Among the patients, 64.2% had consistent GG results between in-bore biopsies and RP, whereas 28.4% had upgraded and 7.4% had downgraded results. GG1 biopsy results, lower biopsy core count, and fewer positive cores were correlated with upgrades in the entire patient group. In patients with $\hbox {GG}>1$, larger tumor sizes and fewer biopsy cores were associated with upgrades. By integrating MRGB data with prebiopsy clinical data, machine learning (ML) models achieved 85.6% accuracy in predicting upgrades, surpassing the 64.2% baseline from MRGB alone. ML analysis also highlighted the value of the minimum apparent diffusion coefficient ($\hbox {ADC}_{\text{min}}$) for $\hbox {GG}>1$ patients. Incorporation of MRGB results with tumor size, $\hbox {ADC}_{\text{min}}$ value, number of biopsy cores, positive core count, and Gleason grade can be useful to predict GG upgrade at final pathology and guide patient selection for active surveillance.

## Introduction

Prostate cancer is the second most frequent cancer diagnosed in men, and despite many improvements in detection and treatment, it is still one of the leading causes of cancer-related mortality^1^. Histology from needle biopsy is crucial for risk stratification and proper selection of treatment options tailored to the characteristics of each tumor and patient. Historically, samples for histopathology have been obtained by standard transrectal ultrasound (TRUS)-guided biopsy using a systematic scheme. However, TRUS-guided prostate biopsy has long been known to result in undersampling, and the other diagnostic uncertainty of this technique is discordance between needle biopsy and RP histologic grading^2,3^, which might lead to under or overtreatment.

In recent years, mp-MRI and subsequent MRI-targeted biopsy techniques have proven to be a highly accurate pathway for detecting clinically significant PCa while simultaneously decreasing the detection of clinically insignificant cancers^4^. The three common MRI-targeted biopsy techniques include visual registration of MRI images with real-time ultrasound images, software-assisted fusion of MRI images and real-time ultrasound images, and MRI-guided in-bore biopsy. MRI-guided in-bore biopsy technique by using ADC maps for fine manual adjustments during the procedure and real-time feedback for needle placement can help to obtain an adequate fraction of the tumor to reach higher concordance between biopsy and final pathology^5,6^. Although there is increased concordance between MRI-targeted biopsy and final pathology from RP, upgrades of the Gleason grade group (GG) are still important, especially for patients with GG1, who are candidates for active surveillance. Active surveillance is a management strategy for low-risk PCa patients designed to avoid overtreatment and the potential side effects of surgery^7^. High Prostate Imaging Reporting and Data System (PI-RADS) scores and/or large tumor sizes on mp-MRI were reported to be predictive factors of upgrading GG1 lesions^8^.

In this retrospective study, an analysis of the clinical variables of patients who underwent MRI-targeted in-bore biopsy and subsequent RP was conducted, with two primary objectives: (i) to identify the clinical variables that were pertinent to the upgrade of GG in the final pathology and (ii) to investigate the possibility of predicting GG upgrade through the utilization of machine learning methods on an individual patient basis, thereby providing a foundation for personalized treatment planning.

## Methods

### Patients

Following the internal review board approval of Koc University, a retrospective examination was conducted on the datasets of 400 men who underwent mp-MRI and subsequent MRI-targeted in-bore biopsy at American Hospital (Istanbul, Turkiye) between 2012 and 2022, with the high likelihood target, i.e. PI-RADS 4 and 5. The research was carried out adhering to the principles outlined in the Declaration of Helsinki. Among the patients who were diagnosed with PCa, 95 of them (median age 64, range 42-78) underwent RP as a definitive treatment. Of these, 20 patients were diagnosed with GG1 PCa based on the in-bore biopsy results. Although GG1 patients normally do not require active treatment and are actively surveilled, the shared decision for definitive treatment took into account the following criteria: History of prostate cancer in the father or brother, International Prostate Symptom Score(IPSS)$>19$, tumor positivity of 2 cores or more, and PI-RADS 4 or PI-RADS 5 lesions bigger than 10 mm. The time interval between in-bore biopsy and RP was less than 6 months for most of the patients. None of the patients had received either radiotherapy or hormone therapy before RP. All biopsy cores and radical prostatectomy specimens were evaluated by a dedicated uropathologist with 16 years of experience, according to the recommendation of the International Society of Urological Pathology (ISUP).

Our study focused on per index lesion level. All high-likelihood lesions (PI-RADS 4 and 5) detected on mp-MRI were targeted by in-bore biopsy. Index lesions were depicted according to PI-RADS version 2 guideline^9^. All index lesions detected on mp-MRI and sampled by in-bore biopsy were confirmed at whole-mount step-section specimens after RP. Since non-index lesions were not clinically related to patient outcomes, they were not analyzed^10–13^.

### Multiparametric MRI and measurements

All multiparametric-MRI examinations were conducted on a 3.0 Tesla MRI Scanner (Magnetom Skyra, Siemens AG, Germany) with sixteen-channel body coil. Butlyscopolamine were used to suppress bowel peristalsis during the examination. The MRI protocol included T2-weighted imaging in axial, coronal and sagittal planes, diffusion-weighted imaging (DWI), and dynamic contrast-enhanced pulse sequences (Table 1).

**Table 1:** Multiparametric MRI protocol.

Parameter	Axial **T2-WI TSE**	Sagittal **T2-WI TSE**	Coronal **T2-WI TSE**	DWI	DCE	T1-WI
TR (ms)	5290	5670	3800	4800	9	445
TE (ms)	111	113	105	98	1.76	9.80
Field of view (mm)	$200 \times 200$	$200 \times 200$	$200 \times 200$	$260 \times 260$	$260 \times 260$	$300 \times 400$
Matrix size	$512 \times 297$	$320 \times 224$	$384 \times 230$	$192 \times 154$	$192 \times 155$	$384 \times 297$
Slice thickness (mm)	3/0.6	2.5/0	2.5/0	3.6/0	3.6/0	6/1.2
Flip angle (^∘^)	180	180	180	180	15	120
Scan time (mins)	03:23	02:46	02:11	05:38	04:38	1:16
Temporal res. (s)	–	–	–	–	8	–
B value ($\hbox {s/mm}^2$)	-	–	–	1600	–	–

**Figure 1:**
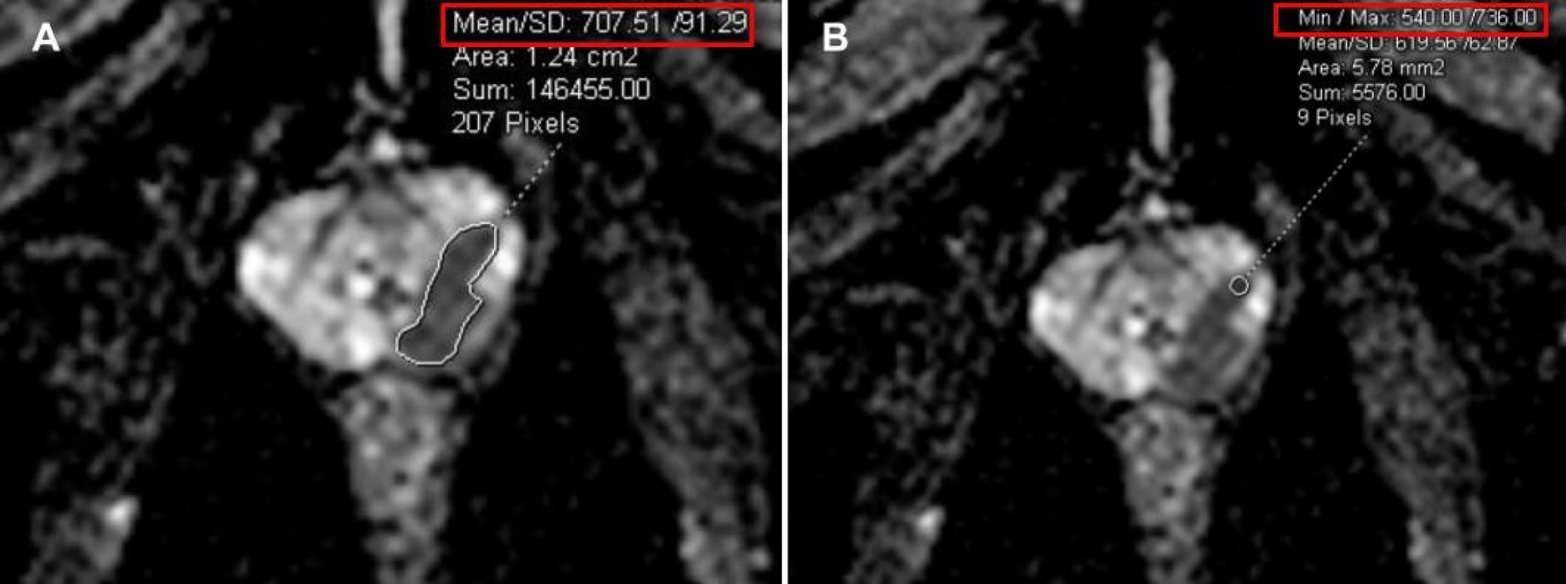
ADC images demonstrate a PI-RADS 5 lesion in the left peripheral zone, which was subsequently confirmed as prostate cancer (Gleason grade 3). The mean ADC and minimum ADC values were measured as shown in Figure (**A**) and (**B**), respectively.

Tumor size, tumor location and PI-RADS scores were interpreted in consensus by three radiologists. ADC values were measured by two radiologists who were blinded to clinical variables and pathology results. The $\hbox {ADC}_{\text{mean}}$ values were obtained by drawing a regions of interest (ROIs) that cover the largest tumor area excluding the tumor edges. While the $\hbox {ADC}_{\text{min}}$ values were obtained by drawing an ROI on the area that visually depicts the lowest ADC value within the tumor (Fig. 1). The interobserver agreement regarding the ADC measurements was assured with 79.3% and 67.7% correlations for mean and min values, respectively. The pathologic interpretation was the same as our previous publication^14^.

### In-bore biopsy technique

In-bore biopsy was performed in an outpatient setting on the same 3 T MRI scanner. All biopsy procedures were carried out by a single radiologist [M.V.] who had more than 15 years of experience in urogenital radiology and interventions.

During the biopsy, the patients were positioned in the prone position. The needle guide, lubricated with 2% lidocaine gel, was inserted into the rectum and attached to a commercially available biopsy device (DynaTRIM, Invivo). To adjust the needle guide placement, sagittal T2W turbo spin-echo images were acquired and transferred to a workstation (DynaCAD, Invivo) in the first place. Subsequently, the software then calculated the target’s rough coordinates relative to the needle guide’s tip, which was manually adjusted toward the target. ADC maps were also utilized during the manual needle adjustments to guide the needle to the area with the lowest ADC values (Fig. 1).

**Figure 2:**
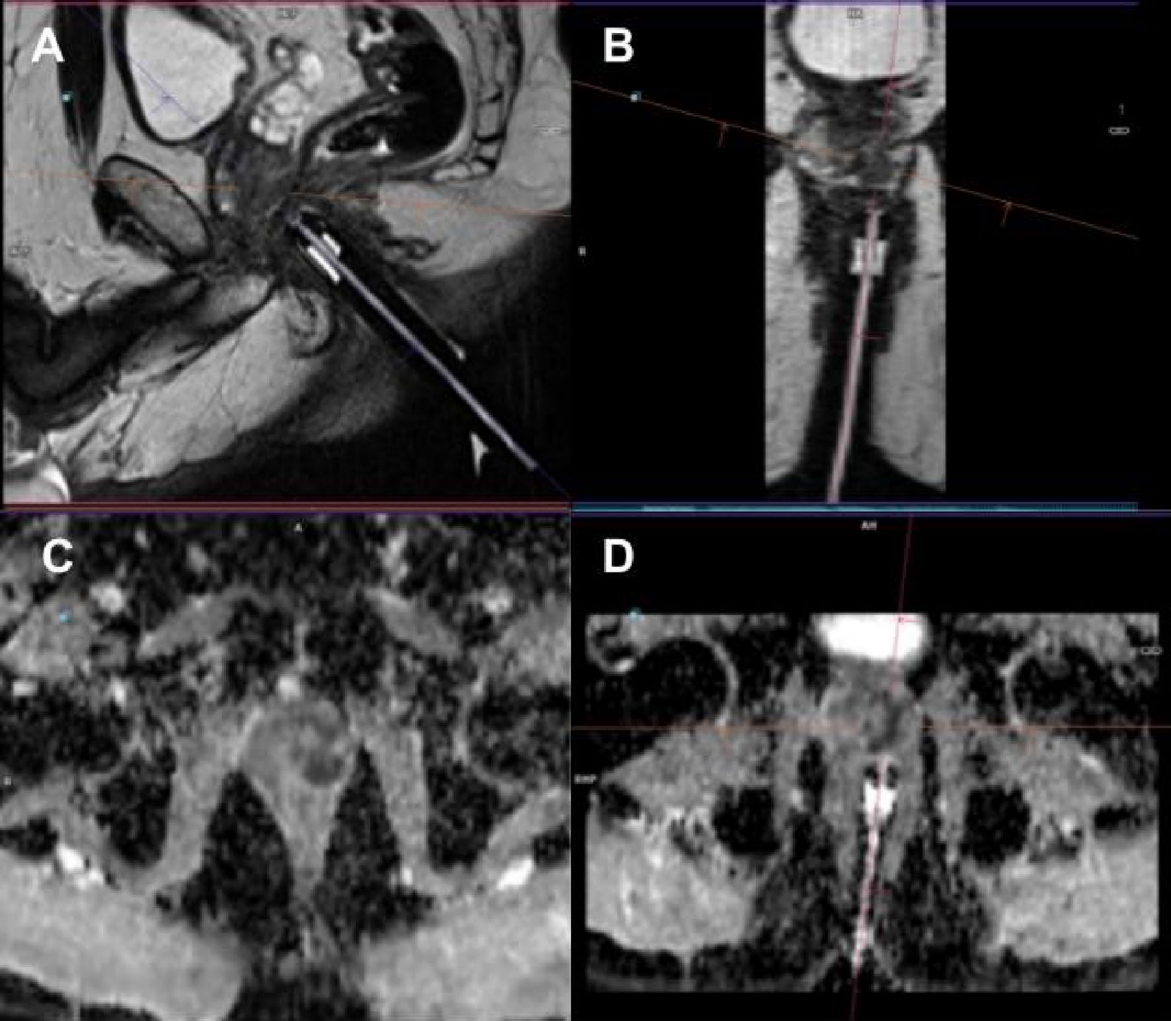
A 57-year-old patient presented with an elevated level of prostate-specific antigen (PSA) measuring 8.9 ng/ml, accompanied by suspicious findings on digital rectal examination. Multiparametric magnetic resonance imaging (mp-MRI) identified a PI-RADS 5 lesion in the left peripheral zone. Subsequently, an MRI-guided in-bore biopsy was performed and the diagnosis of prostate cancer (Gleason Group 4) was established. Sagittal (**A**) and axial (**B**) T2-weighted images, axial ADC image (**D**) showing biopsy needle positioning. Axial ADC image (**C**) taken during in-bore biopsy.

Following the initial adjustments, repeat sagittal and multiplanar reconstructed axial and coronal T2-weighted images were obtained for further fine manual adjustments until the needle guide was accurately pointed to the designated target (Fig. 2). Biopsy cores were obtained using an MRI-compatible, 18-gauge biopsy gun with needle lengths of 150 or 175 mm (In vivo, Gainesville, FL). To ensure accurate sampling of the targeted lesion, the fired needle was left deployed in the prostate, and sagittal and reconstructed T2-weighted images were acquired. Only the suspicious target detected on pre-biopsy mpMRI was sampled without performing a complementary systematic biopsy. During the course of our study, we increased the number of biopsy cores in relation to the growing evidence that focal saturation can improve the compatibility of needle biopsy with whole-mount specimen pathology. On a case-by-case basis, the number of biopsy cores was also affected by the patient’s comorbidities, the history of previous negative biopsy, the size and location of the target, and feedback from needle-in images. The number of biopsy cores that were obtained per each lesion ranged from 2 to 5.

### Clinical parameters

Pre-biopsy clinical variables include patient age, prostate volume, prostate specific antigen (PSA), PSA density (PSAD), tumor size, tumor location (either in peripheral zone (PZ) or transition zone (TZ)), assigned PI-RADS score, mean and minimum ADC values acquired by diffusion weighted images. Biopsy records include number of biopsy cores, number of positive biopsy cores, the ratio of positive cores to total number of cores, total biopsy core length (CL), total biopsy tumor length (TL), TL/CL ratio, and biopsy-assigned GG. Table 2 shows the characteristics of the patients that are involved in this study.

**Table 2:** Patient characteristics comparison for GG upgraded vs non-upgraded cohorts.

Continuous features (unit)	All (N=95)	Upgrade (N=27)	No upgrade (N=68)
	Mean (Std)		
	Median [range (IQR)]		
Age (years)	63.0 (6.6)	61.4 (7.9)	63.6 (5.9)
	64 [42-78 (58-67)]	61 [42-72 (56-67)]	64 [53-78 (59-67)]
PSA (ng/ml)	6.5 (4.3)	5.4 (3.1)	7.0 (4.7)
	5.4 [1.5-26.0 (4.0-7.0)]	5.0 [1.5-16.0 (3.9-6.0)]	5.7 [2.1-26.0 (4.1-7.2)]
Prostate volume (ml)	46.8 (22.6)	46.0 (20.9)	47 (23.5)
	42.0 [16.0-151.0 (30.0-58.0)]	48 [19-110 (28.2-56.9)]	42 [16-151 (30.7-58)]
PSAD (ng/ml/ml)	0.15 (0.11)	0.14 (0.07)	0.15 (0.12)
	0.12 [0.00-0.71 (0.09-0.17)]	0.14 [0.04-0.27 (0.09-0.18)]	0.12 [0.0-0.71 (0.09-0.16)]
$\hbox {ADC}_{\text{mean}}$	774 (184)	769 (190)	776 (182)
	754 [322-1203 (646-882)]	759 [322-1129 (691-877)]	742 [401-1203 (631-882)]
$\hbox {ADC}_{\text{min}}$	634 (163)	639 (199)	632 (148)
	639 [185-955 (531-772)]	664 [185-934 (550-782)]	628 [350-955 (524-712)]
Tumor size (mm)	12.3 (6.7)	14.7 (8.8)	11.4 (5.4)
	11.0 [4.0-40.0 (8.0-15.0)]	12.0 [5-40 (9-19.5)]	11 [4-33 (7-13)]
Total biopsy core length (mm)	41.1 (15.0)	38.8 (15.8)	42.0 (14.7)
	40 [10-79 (31-52)]	36 [12-73 (27-48.5)]	40 [10-79 (32.5-50.5)]
Total biopsy tumor length (mm)	20.6 (12.1)	17.7 (10.9)	21.8 (12.4)
	18 [0.3-66 (12.2-26)]	16 [0.3-40 (11.5-23)]	19 [3-66 (13-27.5)]
TL/CL	0.50 (0.23)	0.46 (0.26)	0.52 (0.21)
	0.5 [0.03-1.0 (0.34-0.64)]	0.44 [0.03-0.95 (0.34-0.59)]	0.50 [0.07-1.0 (0.35-0.66)]
Positive biopsy core ratio	0.91 (0.19)	0.90 (0.23)	0.91 (0.18)
	1.0 [0.25-1.0 (1.0-1.0)]	1.0 [0.25-1.0 (1.0-1.0)]	1.0 [0.25-1.0 (0.94-1.0)]

Categorical features	Value(%)		
PI-RADS	4 (62.1%), 5 (37.9%)	4 (48.1%), 5 (51.9%)	4 (67.6%), 5 (32.4%)
Prostate zone	PZ (85.3%), TZ (14.7%)	PZ (85.2%), TZ (14.8%)	PZ (85.3%), TZ (14.7%)
Number of biopsy cores	2 (11.6%), 3 (41.1%)	2 (25.9%), 3 (51.9%)	2 (5.8%), 3 (36.8%)
	4 (42.1%), 5 (5.2%)	4 (22.2%)	4 (50.0%), 5 (7.4%)
Number of positive biopsy cores	1 (6.3%), 2 (15.8%)	1 (11.2%), 2 (29.6%)	1 (4.4%), 2 (10.3%)
	3 (47.4%), 4 (25.3%)	3 (44.4%), 4 (14.8%)	3 (48.5%), 4 (29.4%)
	5 (5.2%)		5 (7.4%)
Biopsy Gleason grade	1 (21.1%), 2 (42.1%)	1 (63.0%), 2 (25.9%)	1 (4.4%), 2 (48.5%)
	3 (20.0%), 4 (12.6%)	3 (7.4%), 4 (3.7%)	3 (25.0%), 4 (16.2%)
	5 (4.2%)		5 (5.9%)

### Statistical and machine learning analysis

In order to identify clinical parameters that are predictive for GG upgrade, univariate statistics and multivariate machine learning (ML) analyses were performed. For the univariate statistical tests logistic regression was employed. Odds ratio (OR) with confidence interval (CI) that excludes 1 and $\hbox {p}<0.05$ are considered significant.

The baseline prediction accuracy was calculated by comparison of in-bore biopsy and radical prostatectomy Gleason grades, which was used as the benchmark to evaluate the performance of ML models. ML studies were conducted by selecting algorithms that are robust to overfitting for relatively small datasets such as ours, namely, support vector machine (SVM) with linear and radial basis function (RBF) kernels, least absolute shrinkage and selection operator (LASSO) regression, and ridge regression. To assess the performances of the ML algorithms, we used sensitivity, specificity, the area under the receiver operator characteristic (ROC) curve (AUC)^15^, and the Youden index^16^ metrics. Our analyses employed 3 different grouping strategies for the patient cohort: (i) we included all patients and studied all patients with a GG upgrade, (ii) we included $\hbox {GG}>1$ patients and studied all patients with a GG upgrade, and (iii) we included all patients and studied only those with clinically significant upgraded cases, from GG1 to $\hbox {GG}>1$.

The evaluation of performance metrics was conducted through a rigorous process involving 100 randomly selected train-test splits across the dataset, ensuring a comprehensive examination of the model’s robustness and consistency. We adhered to a train-test split ratio of 80% for training data and 20% for testing data. Furthermore, to assess the model’s generalizability and mitigate the risk of overfitting, we employed a 3-fold cross-validation strategy.

### Informed consent

This retrospective observational study was approved by our Institutional Review Board and the requirement for informed written consent was waived by the Koc University School of Medicine ethics committee. All experiments including the study protocol study followed approved institutional guidelines.

## Results

In our study cohort, concordance between biopsy and final pathology GG was recorded in 61 (64.2%) patients. Overall upgrading was recorded in 27 (28.4%) patients, whereas 7 (7.4%) patients were downgraded. Six downgrading men were lowered to the preceding GG, whereas a single case was downgraded by 2 Gleason grade groups (from GG4 to GG2). Among 27 upgraded men, 21 (77.8%) patients’ Gleason grade group were increased by 1 grade. Upgrades by 2 (n=3) and 3 (n=3) grades were also observed equally in 6 cases in total. Among 75 men with biopsy $\hbox {GG}>1$, 10 (13.3%) upgraded cases were observed whereas 58 (77.3%) cases were concordant. Table 3 shows GG distribution obtained by in-bore biopsy versus RP where diagonal elements represent concordance. The upper and lower diagonal elements represent the cases with GG upgrades and downgrades, respectively. All of the upgraded cases from clinically insignificant to clinically significant PCa (17.9% in our study cohort) consisted of upgrades from GS 3+3 to 3+4, whereas downgrading to clinically insignificant PCa did not occur. We focused on the statistics of GG upgrade only due to the lack of downgraded cases. Table 2 gives a comparative account of clinical variable characteristics in men whose GG upgraded after RP in comparison to the men whose GG did not upgrade.

**Table 3:**
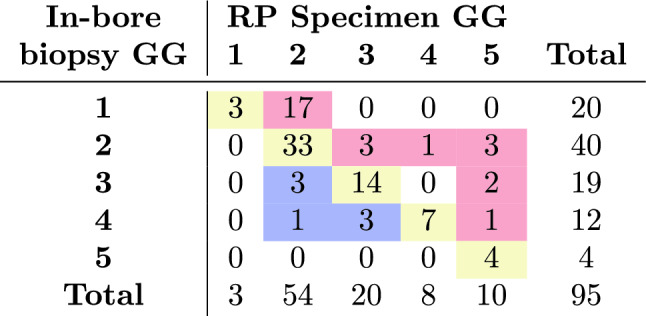
Confusion table of Gleason grades by MRI-guided in-bore biopsy versus RP pathology. Diagonal elements indicate GG concordance. Upper- and lower-diagonal elements indicate GG upgrade and downgrade cases, respectively.

### Statistical analysis

Univariate analyses were conducted using logistic regression, and the results are shown in Table 4. Biopsy GG1 stands out as the most significant predictive factor for a GG upgrade at RP (95% CI 0.06–0.32, $\hbox {p}<0.0001$), such that 17 of the 20 patients with GG1 were upgraded. A smaller number of biopsy cores (95% CI 0.3–0.76, $\hbox {p}=0.002$) and fewer positive biopsy cores (95% CI 0.35–0.87, $\hbox {p}=0.011$) were found to be independent predictive clinical factors by univariate analysis.

The fact that the majority of upgraded patients (17 out of 27) in our study had biopsy GG1 poses the risk that this bulk would saturate our statistics and prevent us from identifying other important upgrade risk factors. Therefore, we repeated the statistical analysis for biopsy $\hbox {GG}>1$ patients only, where increasing tumor size (95% CI 1.07–3.54, $\hbox {p}=0.028$) and decreasing number of biopsy cores (95% CI 0.36–0.97, $\hbox {p}=0.038$) were the statistically significant predictive factors. Furthermore, we studied the clinically significant upgraded cases (from GG1 to $\hbox {GG}>1$), yet none of the clinical parameters turned out to be significant indicators.

The most significant cutoff thresholds for the statistically significant parameters were found by binarizing the parameters using various thresholds and minimizing the p-value. The results indicate that the number of biopsy cores and positive biopsy cores should at least be equal to or larger than 3 and 2, respectively, to decrease the likelihood of GG upgrade. In addition, for the $\hbox {GG}>1$ subgroup, a tumor size equal to 20 mm stands out as the best diagnostic criterion.

**Table 4:** Results of the univariate statistical analysis, given for three different patient groupings: (i) entire cohort, (ii) biopsy $\hbox {GG}>1$ subgroup patients, (iii) the entire cohort where clinically significant upgraded cases (from GG1 to $\hbox {GG}>1$) considered only.

	All patients		Biopsy $\hbox {GG}>1$ patients		Clinically significant GG upgrade cases	
Total population	$\hbox {N}=95$		$\hbox {N}=75$		$\hbox {N}=95$	
Upgraded cases	$\hbox {N}=27$		$\hbox {N}=10$		$\hbox {N}=17$	
Clinical features	OR (CI)	P val.	OR (CI)	P val.	OR (CI)	P val.
Age	0.75 (0.50–1.14)	0.181	1.04 (0.66–1.64)	0.855	0.69 (0.45–1.05)	0.085
PSA	0.75 (0.48–1.17)	0.210	0.90 (0.57–1.42)	0.653	0.80 (0.52–1.23)	0.306
Prostate volume	0.97 (0.65–1.45)	0.866	0.95 (0.61–1.50)	0.841	1.00 (0.67–1.49)	0.994
PSAD	0.92 (0.61–1.38)	0.677	0.99 (0.63–1.56)	0.974	0.91 (0.60–1.36)	0.633
$\hbox {ADC}_{\text{mean}}$	0.98 (0.66–1.46)	0.920	0.80 (0.51–1.28)	0.354	1.22 (0.81–1.83)	0.344
$\hbox {ADC}_{\text{min}}$	1.03 (0.69–1.54)	0.879	0.79 (0.50–1.26)	0.322	1.29 (0.86–1.95)	0.220
Tumor size	1.50 (0.95–2.37)	0.079	1.95 (1.07–3.54)*	0.028*	0.93 (0.62–1.40)	0.734
Total biopsy core length	0.84 (0.56–1.26)	0.407	0.80 (0.51–1.27)	0.351	0.98 (0.65–1.46)	0.907
Total biopsy tumor length	0.76 (0.50–1.15)	0.191	1.00 (0.63–1.57)	0.989	0.71 (0.46–1.09)	0.116
TL/CL	0.82 (0.54–1.23)	0.332	1.11 (0.71–1.75)	0.651	0.72 (0.47–1.09)	0.115
PI–RADS	1.39 (0.92–2.10)	0.112	1.44 (0.91–2.29)	0.121	1.05 (0.70–1.57)	0.813
Tumor zone	1.00 (0.67–1.49)	0.990	1.05 (0.67–1.66)	0.820	0.94 (0.63–1.41)	0.774
Number of biopsy cores	0.48 (0.30–0.76)*	0.002*	0.59 (0.36–0.97)*	0.038*	0.71 (0.47–1.08)	0.111
Number of positive biopsy cores	0.56 (0.35–0.87)*	0.011*	0.69 (0.42–1.11)	0.121	0.71 (0.47–1.09)	0.115
Positive biopsy core ratio	0.96 (0.64–1.44)	0.858	1.03 (0.66–1.63)	0.881	0.91 (0.61–1.37)	0.649
Biopsy GG 1	0.14 (0.06–0.32)*	<0.001*				

Statistically significant results are marked by an asterisks.

Significant values are in bold.

### Machine learning

**Figure 3:**
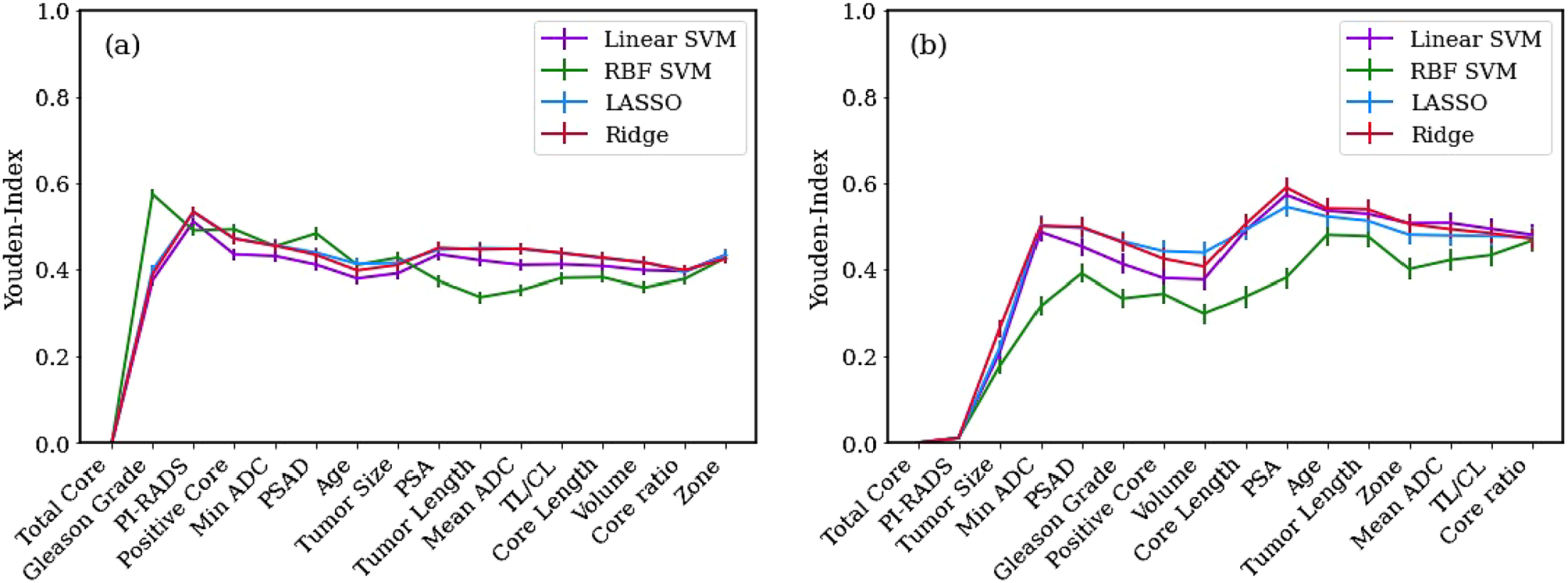
Feature selection by four machine learning models using Youden index as performance metric. Results are shown for (**a**) overall patient cohort and (**b**) only biopsy $\hbox {GG}>1$ group. Error bars indicate standard error of the mean Youden index.

The baseline prediction acuracy set by the in-bore biopsy GG was 64.2% for all patients and 77.3% for the patients with biopsy $\hbox {GG}>1$. We aimed to improve this model by introducing clinical variables. To select the optimum clinical features that maximize the performance of the ML models, we first scaled all clinical variables to the [0, 1] range and then ordered all clinical variables according to their chi-square statistics to GG upgrade. First, machine learning models were trained using only the most correlated feature. Then, at each step, we added the next feature in order and observed its effect on the model performance, measured by the Youden index. At a certain point, the ML models reached a maximum Youden index, and we kept the feature set at that point as our predictive variables. Figure 3a shows the performance of SVM with linear and RBF kernels and LASSO and ridge regressions as a function of the clinical feature set, including the overall patient cohort, after 100 random train-test split iterations. The most favorable results were obtained using an SVM with an RBF kernel (Youden index: $0.575\pm 0.013$, accuracy: $0.856\pm 0.004$, sensitivity: $0.621\pm 0.013$, specificity: $0.953\pm 0.003$, and AUC: $0.865\pm 0.007$) with two predictive clinical features: total number of cores and in-bore biopsy GG.

The same procedure was repeated for biopsy $\hbox {GG}>1$ patients (see Fig. 3b), where ridge regression yielded optimum results (Youden index: $0.590\pm 0.024$, accuracy: $0.904\pm 0.005$, sensitivity: $0.652\pm 0.023$, specificity: $0.938\pm 0.004$, and AUC: $0.944\pm 0.005$) with 10 predictive clinical features, namely, the total number of cores, PI-RADS score, tumor size, $\hbox {ADC}_{\text{min}}$, PSAD, in-bore biopsy GG, number of positive cores, prostate volume, core length, and PSA. The steepest improvement in ML model performances was caused by $\hbox {ADC}_{\text{min}}$ to feature set for $\hbox {GG}>1$ patients. The number of biopsy cores and tumor size also significantly improved the model performance for the entire cohort and biopsy $\hbox {GG}>1$ patients, respectively. Table 5 shows the overall results of the feature selection study with the means and standard errors of the model performance metrics.

**Table 5:** Metrics of the best performing ML models in feature selection.

	AUC	Accuracy	Sensitivity	Specificity	Youden index
(a) All patients					
Linear SVM	$0.859\pm 0.005$	$0.800\pm 0.005$	$0.416\pm 0.013$	$\mathbf{0.958}\pm \mathbf{0.006}$	$0.374\pm 0.013$
RBF SVM	$\mathbf{0.865}\pm \mathbf{0.007}$	$\mathbf{0.856}\pm \mathbf{0.004}$	$\mathbf{0.621}\pm \mathbf{0.013}$	$0.953\pm 0.003$	$\mathbf{0.575}\pm \mathbf{0.013}$
LASSO	$0.859\pm 0.005$	$0.802\pm 0.004$	$0.452\pm 0.014$	$0.946\pm 0.006$	$0.399\pm 0.012$
Ridge	$0.858\pm 0.005$	$0.796\pm 0.005$	$0.449\pm 0.014$	$0.939\pm 0.007$	$0.388\pm 0.012$
(b) Biopsy GG > 1 patients only					
Linear SVM	$\mathbf{0.944}\pm \mathbf{0.004}$	$0.901\pm 0.005$	$0.637\pm 0.023$	$0.936\pm 0.004$	$0.573\pm 0.024$
RBF SVM	$0.894\pm 0.007$	$0.878\pm 0.004$	$0.445\pm 0.024$	$0.936\pm 0.004$	$0.381\pm 0.023$
LASSO	$0.930\pm 0.005$	$0.895\pm 0.005$	$0.613\pm 0.024$	$0.933\pm 0.005$	$0.545\pm 0.023$
Ridge	$\mathbf{0.944}\pm \mathbf{0.005}$	$\mathbf{0.904}\pm \mathbf{0.005}$	$\mathbf{0.652}\pm \mathbf{0.023}$	$\mathbf{0.938}\pm \mathbf{0.004}$	$\mathbf{0.590}\pm \mathbf{0.024}$

The mean scores and their standard deviations of randomly selected 100 train-test splits of (a) all patients and (b) biopsy $\hbox {GG}>1$ patients only.

Significant values are in bold.

**Figure 4:**
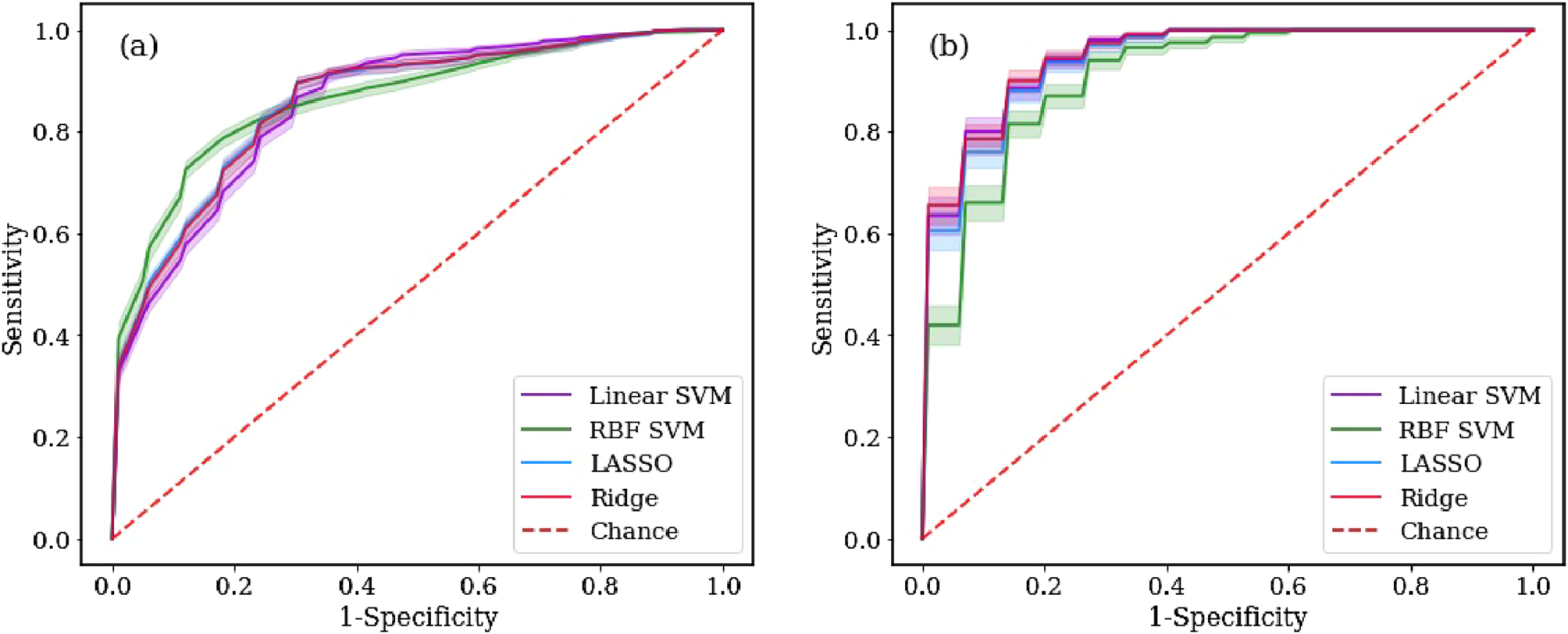
ROC curves for the machine learning models used. Model performances, assessed by AUC metric, using (**a**) overall patient cohort and using (**b**) only biopsy $\hbox {GG}>1$ cases are compared. Shaded regions denote standard error of the mean ROC curves obtained by 100 iterations.

The performance of the machine learning models was also evaluated by receiver operating characteristic (ROC) curves, where the area under the curve (AUC) was used for performance assessment. Figure 4a shows the ROC curves for the four classifier models used. The mean AUC was obtained using 100 random train-test splits on the overall patient group. The RBF SVM model outperformed by achieving AUC: $0.865\pm 0.007$. Figure 4b shows the ROC curves computed from biopsy $\hbox {GG}>1$ patients only. Compared to those of the previous case, the model performances were enhanced. Ridge regression and linear SVM were favored, with an AUC of $0.944\pm 0.004$.

The use of ML algorithms significantly increased the predictability of GGs at RPs. The final pathological GG estimation accuracy of the ML models reached $0.856\pm 0.004$ and $0.904\pm 0.005$ for the entire cohort and biopsy $\hbox {GG}>1$ patient groups, respectively. Compared to the baseline accuracy established by in-bore biopsy alone, these values indicate 21.4% and 13.1% accuracy enhancement for the two cohorts.

## Discussion

Adverse pathology after RP can have serious management consequences, and men with clinically significant disease may be undertreated. Conversely, an overestimated GG would result in overtreatment and hence a reduction in the quality of life of the patient. Therefore, it is of utmost importance to determine the relevant clinical variables that affect GS concordance.

Gleason grade concordance in the literature ranges from 38 to 63%^17–20^. Upgraded cases occur at a rate of 25% to 56%^17–20^, significantly outweighing downgrading cases in the majority of the studies, which range from 8% to 16%^18,19,21^. Although the upgrades in our GG1 group (17 to 20) are remarkable, Costa et al. reported a 66.7% upgrade in the GG1 group^22^. Liu et al. also reported significant upgrade potential in the GG1 subgroup^18^. In our study, the GG upgrade rate was comparable to that in recent studies executed with MRI-targeted biopsy techniques and significantly lower than that in studies with TRUS-guided systematic biopsy^23–25^.

A discordant GG between needle biopsy and final pathology is associated with interobserver variability among different pathologists, borderline grades, and more significantly sampling errors^26^. In support of these arguments, Maruyama et al. reported that GG concordance improved by 6.2% after second-opinion pathology^27^.

In the literature, multiple clinical variables are reported to be predictive for GG discordance, where GG upgrade indicators include older age^20^, higher PSA^20^, lower prostate volume^28^, higher PSAD^28^, higher PI-RADS score^27^, and higher tumor percentage in biopsy cores^29^.

In our study, univariate analysis revealed that age, prostate volume, PSA, PSAD, PI-RADS score, total biopsy core length, total biopsy tumor length, and tumor percentage in biopsy cores were nonsignificant variables for GG upgrade, whereas the number of biopsy cores, number of positive biopsy cores, Gleason grade, and tumor size were found to be significant predictors of GG upgrade. Although the $\hbox {ADC}_{\text{mean}}$ and the $\hbox {ADC}_{\text{min}}$ values were found to be irrelevant variables for GG upgrades in univariate analysis, in the $\hbox {GG}>1$ patient group, multivariate machine learning analysis found the $\hbox {ADC}_{\text{min}}$ value as a useful variable for predicting GG upgrades.

Although the diagnosis of PCa is shifting to targeted biopsy, no agreement has been reached on optimum number of cores. Recent studies showed that more than two biopsy cores had no incremental value in determining the GG^30,31^, however there are some contrary publications suggesting that additional cores from sextants adjacent to designated target (so called focal saturation) can increase biopsy yield and the concordance between needle and final pathology by excluding the effect of GS heterogeneity^32^. According to Tracy et al. the likelihood of GG upgrade decreases with an increase in the number of targeted cores^33^. Our study results also revealed inverse correlation between number of total and positive cores and GG upgrade likelihood at final pathology. Compared to our study with corresponding 28.4% and 17.9% rates, and $3.4\pm 0.8$ cores taken, Ahdoot et al. reported 30.9% and 8.7% rates and Costa et al. reported 13% and 4.4% rates for any GG upgrading and clinically significant upgrading at final pathology with $5.8\pm 2.7$ and average 3.2 MRI-targeted cores, respectively^22,23^.

Intratumoral heterogeneity of tumors is a well known concept and increase in fraction of heterogeneous genetic fusion parallel to tumor size is reported in prostate cancer^34^. Langer et al. showed that peripheral zone prostate cancer is heterogeneous in nature and 36% percent of tumors consists of scattered few malignant glands intermixed with healthy tissue and classified as sparse tumors^35^. To our knowledge, the effect of tumor size on GG upgrade was not studied in literature. Our study showed that tumors with larger sizes were upgraded more than tumors with smaller size, which is statistically significant for $\hbox {GG}>1$ subgroup ($\hbox {p}=0.028$). Our analysis revealed 20 mm as a threshold for $\hbox {GG}>1$ group, and showed that tumors over 20 mm have a higher possibility to upgrade after biopsy. Due to size criteria of PI-RADS 2.1^36^, our threshold with 20 mm falls into PI-RADS 5 category. The correlation between PI-RADS scores and Gleason grades is well known^37–39^, besides that in accordance with our results, the correlation with upgrades of GG and PI-RADS score was demonstrated by Alqahtani et al.^40^. Meta-analysis about active surveillance stated precautious results with active surveillance of PI-RADS 4 and 5, which can be related to our finding with high upgrade ratios in GG1 group^41^. In addition to that, our model stated that tumor size has a value for predicting upgrade after in-bore prostate biopsy, which is a novel finding. This finding, if supported by future research with larger series, may have important implications for clinical practice, including considering focal saturation in tumors with large dimensions.

Diffusion weighted imaging is a key component of mp-MRI that contributes to tumor detection, as well as to the assessment of tumor aggressiveness. Tissue microstructure such as dense cellularity or atrophic glands can result in distinct imaging findings. Hambrock et al. showed a high discriminatory performance can be achieved in the differentiation of low, intermediate, and high-grade PCa by ADC value^42^. In active surveillance patient group, ADC value was identified as an independent predictor of both upgrading on repeat biopsy and time to radical therapy^26,43,44^. Park et al. reported a significant inverse correlation between the $\hbox {ADC}_{\text{mean}}$ and the $\hbox {ADC}_{\text{min}}$ values and the possibility of GG upgrade in a patient group of GG1^45^. In our study, statistical analysis revealed no significant correlation between the $\hbox {ADC}_{\text{mean}}$ and $\hbox {ADC}_{\text{min}}$ values and GG upgrade in both groups of patients whereas in ML studies $\hbox {ADC}_{\text{min}}$ value was found to be useful in the prediction of GG upgrade in the $\hbox {GG}>1$ patient group. This discrepancy between $\hbox {ADC}_{\text{min}}$ and $\hbox {ADC}_{\text{mean}}$ can be explained by the heterogeneous nature of PCa.

Various ML algorithms previously used for GG upgrade prediction are logistic regression^18^, LASSO regression^18,46^, SVM^18,47^, k-Nearest Neighbours (kNN)^46^, decision trees^46^, and random forests^18,46^. Due to the lack of large datasets, medical problems pose a particular challenge for ML models. Many machine learning algorithms require a considerable amount of data. Otherwise, the ML model may overfit the training data and generate poor results on the tests. For this reason, ML models such as decision tree and random forest that require massive datasets are not suitable candidates for our problem, agreed by the former studies in literature^18,46^. SVM, Ridge and LASSO models were used in this study as they are less prone to overfitting for relatively small datasets.

Our results show ML-assisted GG estimation accuracy was increased by 21.4% for the overall patient group, surpassing the 13.1% enhancement for upgrade estimations among $\hbox {GG}>1$ cases, in line with the literature where Liu et al.^18^ showed ML application improved the prediction accuracy from 39.2% to 71.2%. These accuracy enhancements indicate ML models are useful tools to utilize clinical records for personalized treatment planning. Moreover, ML models unraveled the significance of more clinical features than revealed by statistics alone such as $\hbox {ADC}_{\text{min}}$ (see Fig. 3b), outlining the power of ML concept, where features considered statistically insignificant can be utilized for predictive models.

The potential limitations of this study are retrospective design, small sample size that affects both statistical and ML studies, and possible increase in selection bias due to recruitment of patients over 8 years of time. Biopsy GG1 patients upgraded at RP pathology more often compared to other biopsy Gleason grade groups. The reason for this may be due to bias in data collection, as most low-risk GG1 patients are assigned to active surveillance rather than RP. Additionally, even though a 3-fold cross-validation strategy was employed in our study, an external validation is crucial for confirming the model’s effectiveness and applicability in different clinical settings. Future studies should aim to incorporate such validation to ensure the model’s reliability and utility in the clinical management of prostate cancer, enhancing its potential contribution to personalized patient care.

Our study pioneers the application of machine learning methodologies to predict upgrades in MRI-guided in-bore biopsy patients, boasting the second-largest study population, which compares MRI-guided in-bore biopsy and radical prostatectomy results^48^. Overall, our study suggests that a combination of clinical factors (the number of biopsy cores, the number of positive biopsy cores, Gleason grade, tumor size and $\hbox {ADC}_{\text{min}}$ value) and machine learning models may be valuable in predicting the likelihood of GG upgrade following RP and could potentially improve patient outcomes.

## Conclusion

Determining the relevant clinical variables that affect GS concordance in MRI-targeted biopsy is of utmost importance in the era of MRI pathway. Univariate statistics revealed the number of biopsy cores, number of positive biopsy cores, and Gleason grade were statistically significant GG upgrade indicators and inversely correlated to GG upgrade possibility. Machine learning analysis found the $\hbox {ADC}_{\text{min}}$ value as a useful variable in the prediction of GG upgrade. As a novel finding, tumor size measured by mpMRI is shown to be positively correlated with GG upgrade likelihood for $\hbox {GG}>1$ subgroup. Tumor size and $\hbox {ADC}_{\text{min}}$ can be useful markers to assess risk of upgrade prior to biopsy, so biopsy number and patient selection for active surveillance can be decided in terms of these markers. The findings of our study contribute to identifying patients predisposed to GG upgrade during RP. By comparing patient characteristics with our documented outcomes, we can pinpoint high-risk cases for GG upgrade and potentially adjust the threshold for performing RP in such cases.

### Supplementary Information

Supplementary Information.

## Data Availability

The data that support the findings of this study are available upon reasonable request from the corresponding author.
