# Supplementary material for: Machine learning prediction of Gleason grade group upgrade between in-bore biopsy and radical prostatectomy pathology
Source: Sci Rep. 2024 Mar 11;14:5849. doi: 10.1038/s41598-024-56415-5 (PMC10925603; doi:10.1038/s41598-024-56415-5)
Supplement: Supplementary file 1 — Supplementary Information. [file 41598_2024_56415_MOESM1_ESM.pdf]

Supplementary document for "Machine learning prediction of Gleason grade group upgrade between in-bore biopsy and radical prostatectomy pathology"

Kaan Ozbozduman*[1], Irem Loc*[1], Selahattin Durmaz[2], Duygu Atasoy[3], Mert Kilic[4], Hakan Yildirim[5], Tarik Esen[4,6], Metin Vural[5], and M. Burcin Unlu[7,8]

[1] *Bogazici University Physics Department, Istanbul, Turkey*
[2] *Department of Radiology, Istanbul Faculty of Medicine, Istanbul University, Istanbul, Turkey*
[3] *Department of Radiology, University of Koc School of Medicine, Istanbul, Turkey*
[4] *Department of Urology, VKF American Hospital, Istanbul, Turkey*
[5] *Department of Radiology, VKF American Hospital, Istanbul, Turkey*
[6] *Department of Urology, University of Koc School of Medicine, Istanbul, Turkey*
[7] *Faculty of Engineering, Ozyegin University, Istanbul, Turkey*
[8] *Faculty of Aviation and Aeronautical Sciences Ozyegin University, Istanbul, Turkey*

As described in the methods section, we employed 3 grouping strategies for univariate analysis: i) we included all patients and studied all patients with a GG upgrade, ii) we included GG>1 patients and studied all patients with a GG upgrade, and iii) we included all patients and studied only those with clinically significant upgraded cases, from GG1 to GG>1."

Patient characteristics for the first group are already given in Table 2. Here, we include two supplementary tables: one for the second (Table 1) and the other for the third (Table 2) group to state the related patient characteristics.

Table 1: Patient characteristics comparison for GG>1 patients where all GG upgrades were studied.

	**All (N=75)**	**Upgrade (N=10)**	**No upgrade (N=65)**
**Continuous features (unit)**	**Mean (Std)**<br>**Median [Range (IQR)]**		
Age (years)	63.8 (5.9)<br>64 [52-78 (59-67)]	64.3 (6.1)<br>67 [52-70 (61-69.25)]	63.8 (5.9)<br>64 [54-78 (59-67)]
PSA (ng/ml)	6.9 (4.6)<br>5.5 [2.1-26.0 (4.3-7.2)]	5.9 (2.7)<br>5.1 [2.5-12.0 (4.9-5.6)]	7.1 (4.9)<br>5.9 [2.1-26.0 (4.1-7.5)]
Prostate volume (ml)	46.7 (22.8)<br>43.0 [16.0-151.0 (30.0-57.5)]	44.5 (15.6)<br>49 [20-67 (31.5-55.5)]	47.1 (23.9)<br>42 [16-151 (30.0-58.0)]
PSAD (ng/ml/ml)	0.2 (0.1)<br>0.13 [0.0-0.71 (0.09-0.18)]	0.1 (0.1)<br>0.15 [0.04-0.25 (0.09-0.21)]	0.2 (0.1)<br>0.12 [0.0-0.71 (0.09-0.16)]
ADCmean	750.8 (171.1)<br>725 [401-1203 (628-847)]	683.5 (145.5)<br>687 [415-864 (635-809)]	763.0 (173.7)<br>731 [401-1203 (624-860)]
ADCmin	616.8 (154.9)<br>618 [185-955 (517-683)]	555.2 (174.1)<br>612.5 [185-721 (469-674)]	628.1 (150.2)<br>618 [350-955 (519-697)]
Tumor size (mm)	12.4 (6.8)<br>11.0 [4.0-40.0 (8.0-15.0)]	18.7 (10.0)<br>15.5 [8.0-40.0 (12.7-20.7)]	11.2 (5.4)<br>11.0 [4.0-33.0 (7.0-13.0)]
Total biopsy core length (mm)	41.6 (14.2)<br>40.5 [10-79 (32-48.75)]	35.9 (11.4)<br>36.5 [12.0-50.0 (30.5-44.25)]	42.5 (14.5)<br>40.5 [10-79 (33-49)]
Total biopsy tumor length (mm)	22.3 (12.5)<br>19.0 [0.3-66.0 (14.0-29.5)]	22.2 (13.9)<br>18.5 [0.3-40.0 (14.0-36.5)]	22.4 (12.4)<br>20.0 [3.0-66.0 (14.7-28.5)]
TL/CL	0.5 (0.2)<br>0.5 [0.03-1.0 (0.34-0.69)]	0.6 (0.3)<br>0.615 [0.03-0.95 (0.35-0.81)]	0.5 (0.2)<br>0.5 [0.07-1.0 (0.35-0.65)]
Positive biopsy core ratio	0.9 (0.2)<br>1.0 [0.25-1.0 (1.0-1.0)]	0.9 (0.2)<br>1.0 [0.33-1.0 (1.0-1.0)]	0.9 (0.2)<br>1.0 [0.25-1.0 (1.0-1.0)]
**Categorical features**	**Value(%)**		
PI-RADS	4 (62.7%), 5 (37.3%)	4 (30.0%), 5 (70.0%)	4 (67.7%), 5 (32.3%)
Prostate zone	TZ (13.3%), PZ (86.7%)	TZ (10.0%), PZ (90.0%)	TZ (13.8%), PZ (86.2%)
Number of biopsy cores	2 (8%), 3 (42.7%)<br>4 (42.7%), 5 (6.7%)	2 (20%), 3 (80%)	2 (6.2%), 3 (36.9%)<br>4 (49.2%), 5 (7.7%)
Number of positive biopsy cores	1 (4%), 2 (12%), 3 (50.7%)<br>4 (26.7%), 5 (6.7%)	1 (10%), 2 (20%), 3 (70%)	1 (3.1%), 2 (10.8%)<br>3 (47.7%), 4 (30.8%)<br>5 (7.7%)
Biopsy Gleason grade group	2 (53.3%), 3 (25.3%)<br>4 (16%), 5 (5.3%)	2 (70%), 3 (20%)<br>4 (10%)	2 (50.8%), 3 (26.2%)<br>4 (16.9%), 5 (6.2%)

Table 2: Patient characteristics comparison for all patients where only those with clinically significant upgraded cases were studied.

	**All (N=95)**	**Upgrade (N=27)**	**No upgrade (N=68)**
**Continuous features (unit)**	**Mean (Std)**<br>**Median [Range (IQR)]**		
Age (years)	63.0 (6.6)<br>64.0 [42.0-78.0 (58.0-67.0)]	59.8 (8.5)<br>59.0 [42.0-72.0 (54.0-67.0)]	63.7 (5.9)<br>64.0 [52.0-78.0 (59.2-67.0)]
PSA (ng/ml)	6.5 (4.3)<br>5.4 [1.5-26.0 (4.0-7.0)]	5.2 (3.4)<br>4.0 [1.5-16.0 (3.4-6.0)]	6.8 (4.5)<br>5.45 [2.1-26.0 (4.2-7.1)]
Prostate volume (ml)	46.8 (22.6)<br>42.5 [16.0-151 (30.0-58.0)]	47.1 (24.5)<br>46.5 [19.0-110 (27.5-58.7)]	46.7 (22.4)<br>42.5 [16.0-151 (30.2-57.5)]
PSAD (ng/ml/ml)	0.2 (0.1)<br>0.125 [0.0-0.71 (0.09-0.17)]	0.1 (0.1)<br>0.115 [0.06-0.27 (0.09-0.16)]	0.2 (0.1)<br>0.125 [0.0-0.71 (0.09-0.17)]
ADCmean	774.2 (183.7)<br>754.5 [322-1203 (646-882)]	831.6 (198.5)<br>844 [322-1129 (729-934)]	762.4 (179.8)<br>730 [401-1203 (631-854)]
ADCmin	634.3 (163.3)<br>639 [185-955 (531-722)]	698.9 (199.5)<br>734 [307-934 (575-864)]	621.0 (153.1)<br>628.5 [185-955 (520-710)]
Tumor size (mm)	12.3 (6.7)<br>11.0 [4.0-40.0 (8.0-15.0)]	11.7 (6.8)<br>10.0 [5.0-25.0 (7.0-12.0)]	12.4 (6.7)<br>11.0 [4.0-40.0 (8.0-15.0)]
Total biopsy core length (mm)	41.1 (15.0)<br>40.0 [10.0-79.0 (31.0-50.0)]	40.6 (18.1)<br>36.0 [14.0-73.0 (27.0-54.0)]	41.2 (14.4)<br>40.0 [10.0-79.0 (32.0-49.0)]
Total biopsy tumor length (mm)	20.6 (12.1)<br>18.0 [0.3-66.0 (12.25-26.0)]	15.1 (8.1)<br>16.0 [1.0-28.0 (9.0-21.0)]	21.9 (12.5)<br>19.0 [0.3-66.0 (13.0-28.0)]
TL/CL	0.5 (0.2)<br>0.5 [0.03-1.0 (0.34-0.64)]	0.4 (0.2)<br>0.38 [0.05-0.8 (0.33-0.58)]	0.5 (0.2)<br>0.5 [0.03-1.0 (0.34-0.7)]
Positive biopsy core ratio	0.9 (0.2)<br>1.0 [0.25-1.0 (1.0-1.0)]	0.9 (0.2)<br>1.0 [0.25-1.0 (1.0-1.0)]	0.9 (0.2)<br>1.0 [0.25-1.0 (1.0-1.0)]
**Categorical features**	**Value (%)**		
PI-RADS	4 (62.1%), 5 (37.9%)	4 (58.8%), 5 (41.2%)	4 (62.8%), 5 (37.2%)
Prostate zone	PZ (85.3%), TZ (14.7%)	PZ (82.4%), TZ (17.6%)	PZ (85.9%), TZ (14.1%)
Number of biopsy cores	2 (11.6%), 3 (41.1%)<br>4 (42.1%), 5 (5.2%)	2 (29.4%), 3 (35.3%)<br>4 (35.3%)	2 (7.7%), 3 (42.3%)<br>4 (43.6%), 5 (6.4%)
Number of positive biopsy cores	1 (6.3%), 2 (15.8%)<br>3 (47.4%), 4 (25.3%)<br>5 (5.2%)	1 (11.8%), 2 (35.3%)<br>3 (29.4%), 4 (23.5%)	1 (5.1%), 2 (11.5%)<br>3 (51.3%), 4 (25.6%)<br>5 (6.4%)
Biopsy Gleason grade	1 (21.1%), 2 (42.1%)<br>3 (20.0%), 4 (12.6%)<br>5 (4.2%)	1 (100%)	1 (3.8%), 2 (51.3%)<br>3 (24.4%), 4 (15.4%)<br>5 (5.1%)
